# Lateral approach is a more aesthetical option for radical resection of BSCC: assessment of its surgical, oncological, functional, and aesthetic outcomes

**DOI:** 10.1186/s12903-022-02519-1

**Published:** 2022-11-03

**Authors:** Wen-Dong Wan, Can Lu, Yong-Xiang yuan, Jia-Ju Hu, Jie Liang, Cai-Yun He, Yu-Qi Huang, Tong Su, Feng Guo, Can-Hua Jiang, Ning Li

**Affiliations:** 1 Department of Oral and Maxillofacial Surgery, Center of Stomatology, Xiangya Hospital, Central South University, Changsha, China; 2 Institute of Oral Precancerous Lesions, Central South University, Changsha, China; 3 Research Center of Oral and Maxillofacial Tumor, Xiangya Hospital, Central South University, Changsha, China; 4 National Clinical Research Center for Geriatric Disorders, Xiangya Hospital, Central South University, Changsha, China

**Keywords:** Buccal cancer, Neck dissection, Combined radical resection, Surgical approach, Aesthetic outcome

## Abstract

**Background:**

The purpose of this study was to introduce a modified lateral approach for combined radical resection of buccal squamous cell carcinoma (BSCC) and evaluate its surgical, oncological, functional, and aesthetic outcomes in comparison with the conventional lower-lip splitting approach.

**Methods:**

This single-center study retrospectively reviewed 80 patients with BSCC, of which 37 underwent the lateral approach and 43 underwent the conventional approach. Surgical, functional, oncological, and aesthetic evaluations, as well as follow-ups, were recorded and compared.

**Results:**

Compared to the conventional approach group, the lateral approach group had a longer surgical time (*P* = 0.000), but there was no significant difference in other surgical and oncological parameters. Moreover, the scar in the head and neck had a significantly discreet appearance in the lateral approach group, whose satisfaction was better than those in the conventional approach group (*P* = 0.000). Other oral function parameters, postoperative mouth-opening, and 3-year survival rate were not significantly different between the two groups.

**Conclusion:**

The lateral approach could provide superior aesthetic results while maintaining equal surgical, functional, and oncological outcomes compared to the conventional approach for radical resection of BSCC.

**Supplementary Information:**

The online version contains supplementary material available at 10.1186/s12903-022-02519-1.

## Background

The incidence of buccal squamous cell carcinoma (BSCC) has been increasing in China and some Asian countries, mainly because of the high prevalence of betel quid chewing [1]. Although significant advances have been made in the prevention, diagnosis, and therapy of oral cancer in recent decades, combined radical resection remains the primary modality for BSCC treatment [2].

Several types of incisions can be used for patients with BSCC; however, in practice, the midline or lateral lower lip-splitting incision has been the most commonly performed to obtain favorable surgical exposure [3]. All lower lip-splitting approaches can result in some adverse aesthetic and functional complications such as facial unsightly scars, vermilion notching, loss of chin pad contour, decreased lip sensation and mobility, and oral commissure incontinence [4]. Although the most important aim of BSCC therapy is to radically remove the mass, the surgical scar left on the exposed neck and face is most likely to decrease the postoperative patient satisfaction. The transoral resection approach for oral and oropharyngeal tumors by robot-assisted and endoscopic surgeries is the least invasive method to avoid postoperative facial aesthetics or functional complications [5]. However, not all clinical institutions have the robot equipment and experience to carry out such an approach. In addition, robot-assisted surgery is currently expensive and requires additional training for surgeons. Thus, several modifications of the lower lip-splitting incision have been proposed to reduce postoperative aesthetic and functional complications, including Roux-Trotter incision, Robson incision, and McGregor incision [6]. However, these modifications can still result in scars on the face and head because the lower lip and chin require to be incised. The only way to avoid postoperative aesthetic and functional complications is to maintain continuity of the lower lip and orbicular muscles.

In addition to its application in plastic surgery, the face-lift approach has been presented for removing masses in the mid-cheek region owing to its obvious advantages of superior facial cosmesis [4]. Moreover, the lateral hockey-stick incision for neck dissection can provide good access to levels I–III and satisfactory cosmetic outcomes [7]. If the lateral hockey-stick incision is combined with the face-lift approach for en bloc resection of BSCC, it may be possible to obtain a more concealed scar while providing good exposure.

In the current study, we used a novel lateral approach for en bloc resection of BSCC, which combined the face-lift and lateral hockey-stick incisions. By comparing this modified approach to the conventional lower lip-splitting incision, we found that the lateral approach was reliable for improving the postoperative aesthetic results of the face and head, decreased the possible functional morbidities of the lower lip, and preserved the oncological goals of the radical resection of BSCC.

## Methods

### Eligibility criteria

**Fig. 1:**
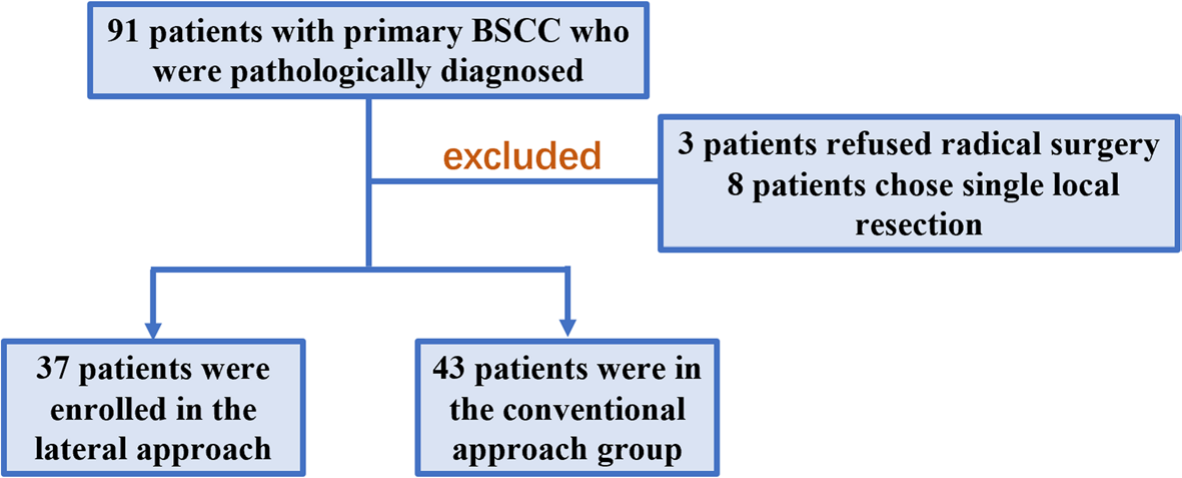
The flow chart of patient selection in the study. A total of 91 patients with primary BSCC were enrolled. Among them, 11 patients were excluded from our study. Finally, 37 were enrolled in the lateral approach group, and another 43 patients were in the conventional approach group

Between January 2018 and April 2020, a total of 91 patients with primary BSCC who were pathologically diagnosed before admission were enrolled from the Department of Oral Maxillofacial Surgery in Xiangya Hospital. Among them, 11 patients were excluded from our study (3 patients refused the radical surgery, and 8 early-stage patients chose local resection). The remaining patients received either the conventional approach with the lower lip-splitting incision or the lateral approach using the modified facelift incision combined with hockey-stick incision for the radical resection of BSCC. Finally, 37 (30 men and 7 women) were enrolled in the lateral approach group, and another 43 patients (32 men and 11 women) were in the conventional approach group. Figure 1 shows the flow chart of patient selection. Informed consent was obtained from all the participants. Patient and tumor characteristics were recorded, including age, sex, T classification, N classification, and TNM stage. Surgical outcome variables were documented, including the type of neck dissection, total operative time, blood loss, postoperative drainage, surgical margin, lymph node retrieval, length of hospital stay (LOHS), and operative complications. All procedures performed in this study were in accordance with the ethical standards of the institutional and/or national research committee and the 1964 Helsinki Declaration and its later amendments or comparable ethical standards. No randomization was performed between the two groups. The decision of skin incision was mainly determined according to the surgeon’s preference and patient’s choice (after patients fully understood the surgical plan and made the incision choice, Dr. Ning performed the lateral incision, and Dr. Canhua and Dr. Feng performed the lower lip-splitting incision).

### Surgical technique

**Fig. 2:**
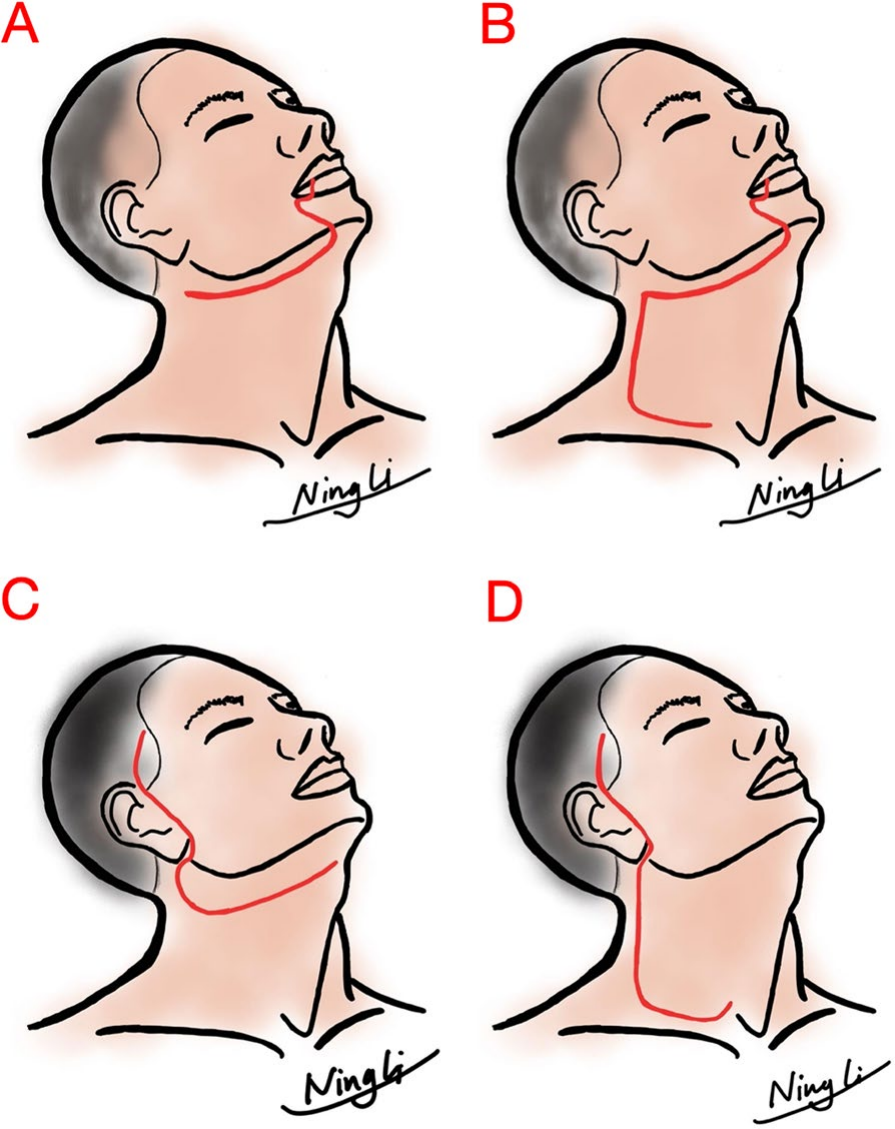
The diagrams of surgical incision for the two approaches. **A,B** The surgical incision of the conventional approach for SND and MRND; **C, D** The surgical incision of the lateral approach for SND and MRND.

In our institution, the conventional incision approach was also described as the para-lower-lip approach by Xian et al. [8]. It began from the oral commissure of the lower lip vermilion, descended along the lateral border of the triangularis muscle to the submental region, and then turned backward 2 cm beneath the inferior edge of the mandible to the mastoid tip for selective neck dissection (SND) (Fig. 2 A), or continued to curve downward to the neck root above the clavicle for the modified radical neck dissection (MRND) (Fig. 2B).

The incision line of the modified lateral approach was made from the sideburn, descended downward along the anterior tragus to the earlobe, and then curved postauricularly to the mastoid tip. For SND, the incision was turned downward along the anterior border of the trapezius to the middle neck and extended transversely along the natural neck crease to the midline (Fig. 2 C). For MRND, after reaching the mastoid tip, the incision line turned downward along the anterior border of the trapezius until 2 cm above the clavicle, and then extended transversely to the midline (Fig. 2D).

**Fig. 3:**
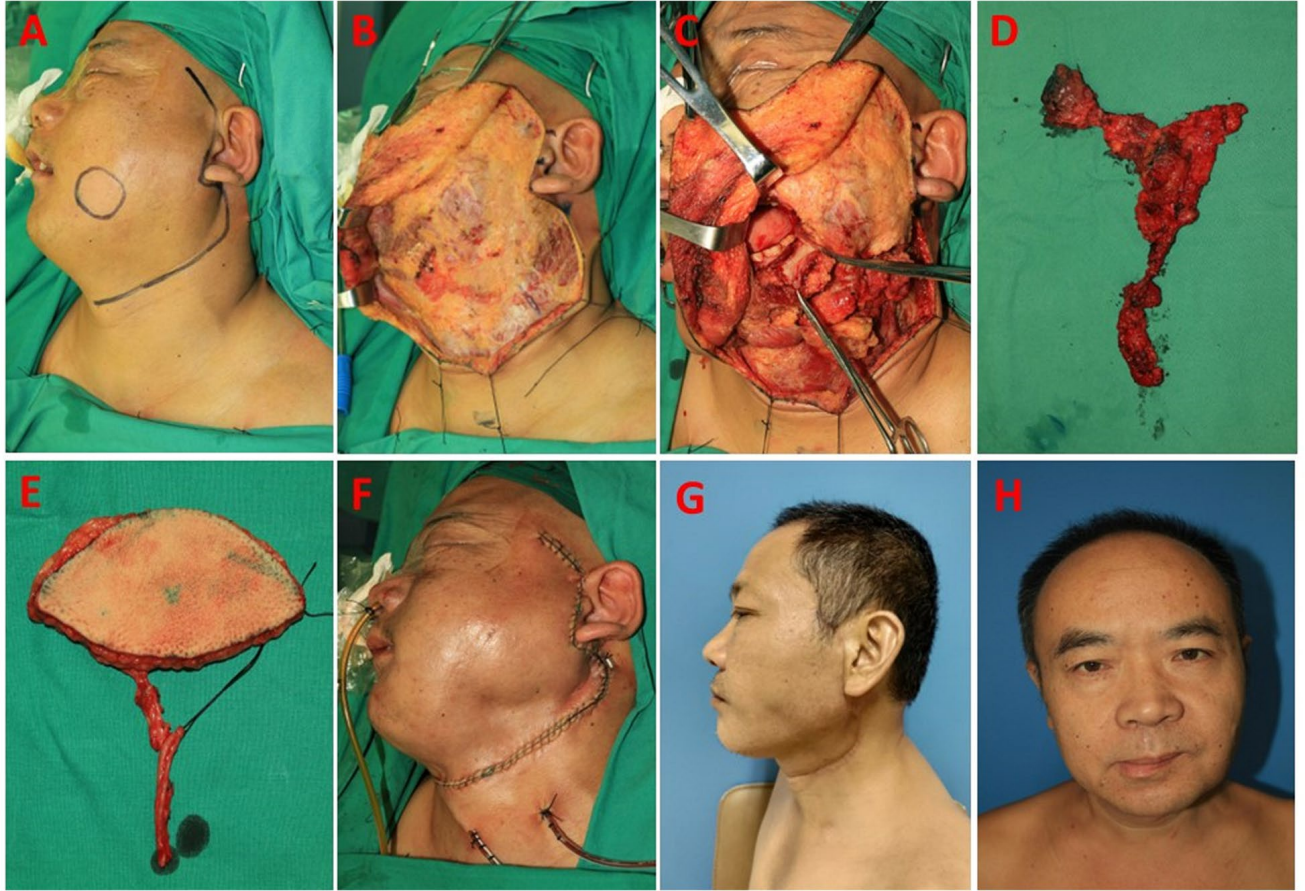
T_2_N_0_M_0_ BSCC case with SND by lateral approach. **A** The lateral approach line for SND; **B** The skin flap was elevated until the submental region; **C** Pulled the tumor out from the oral cavity to check the surgical margins; **D** Primary tumor and neck-dissection tissues were en bloc resected; **E** ALTF for the reconstruction of intraoral defects; **F** Wounds closed primarily; **G,H** Six months after operation

Herein, we consider two cases of BSCC as examples to briefly describe the surgical procedures of the lateral approach. One man with T_2_N_0_M_0_ BSCC received SND. After designing and incising the surgical line (Fig. 3 A), the neck skin flap was elevated under the platysma and then forwarded to the omohyoid muscle, hyoid bone, and region Ia (Fig. 3B). In cases that the buccal skin did not need full-thickness resection, to ensure safe margin of the tumor bottom, when the face-lift flap was elevated near the tumor bottom, we made the elevation plane of face flap more superficial which was between the subcutaneous tissue and the buccinator muscle. Thus, the buccinator muscle of at least 1.5 cm around the tumor bottom should be retained with the tumor. SND was then performed from the bottom to the top under good visualization. When resecting the primary tumor, the upper, anterior, and posterior surgical margins of the buccal tumor were incised first in the full layer of the oral cavity, and the tumor was then pulled out from the oral cavity to check whether the surgical margins of the tumor were safe (Fig. 3 C). After incising the lower margin of the tumor, we performed en bloc resection of the primary tumor and neck dissection tissues (Fig. 3D). The intraoral defect was routinely repaired using free flaps such as the anterior lateral thigh flap (ALTF) (Fig. 3E). All the wounds were closed primarily (Fig. 3 F). All surgical scars were hidden in the lateral head and neck. Six months after the operation, no surgical scar was found on the face of this patient (Fig. 3G,H).

**Fig. 4:**
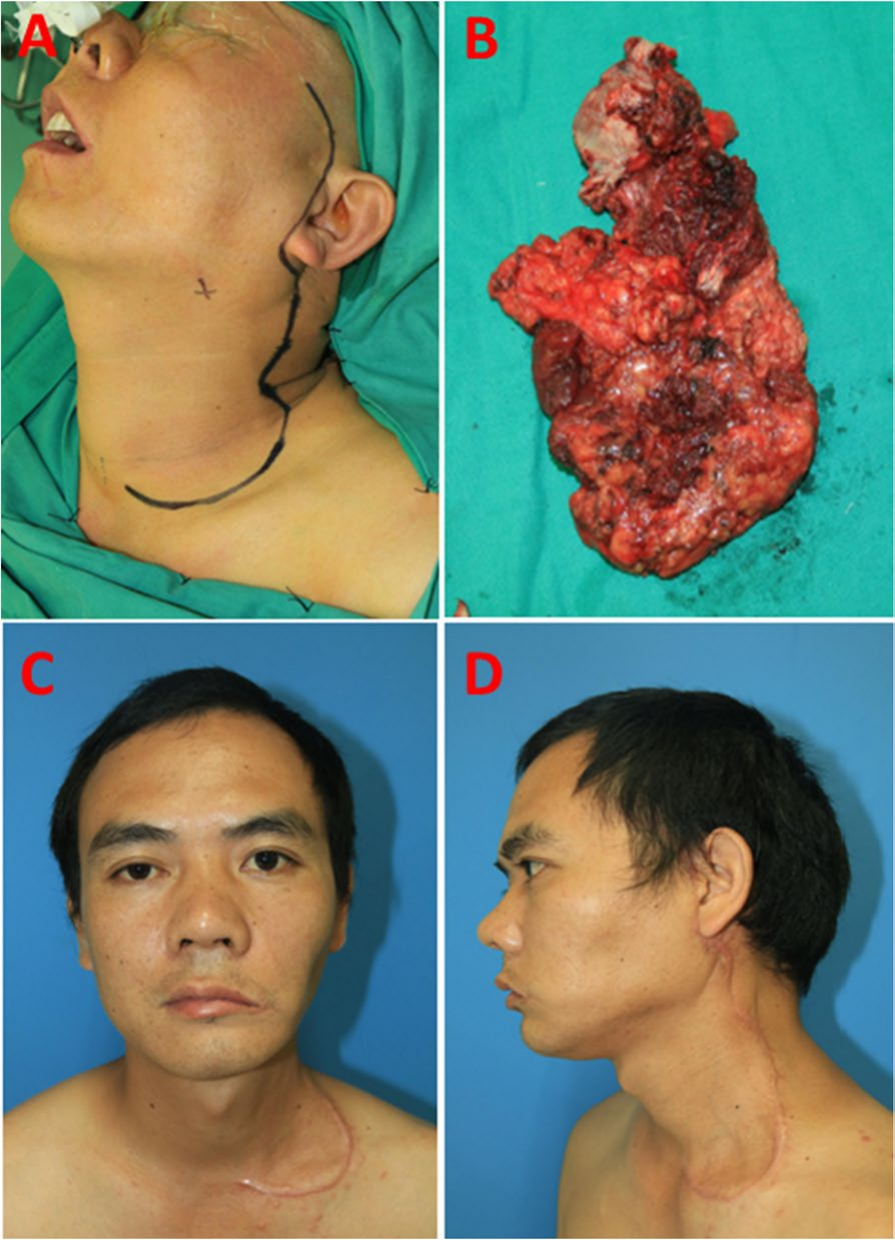
A man with BSCC undergoing MRND. **A** The lateral approach line for MRND; **B** Primary tumor and neck-dissection tissues were en bloc resected; **C,D** Six months after operation

The other patient with T_2_N_1_M_0_ BSCC underwent MRND. After the design of the surgical incision lines (Fig. 4 A), the surgical procedures were similar to those in SND. The primary tumor and neck-dissection tissues were resected en bloc (Fig. 4B). Surgical scars were also hidden in the lateral head and neck rather than in the face six months after the operation (Fig. 4 C,D).

In the present study, we also used the lateral approach for radical resection of advanced T3 or T4 patients whose cheek skin required through-and-through resection. However, the lateral approach did not achieve better aesthetic outcomes in such advanced patients than the conventional approach because both approaches inevitably cause obvious scars on the face (Supplementary Figure).

### Postoperative evaluation and follow-up assessment

Postoperative quality of life assessed using the University of Washington Quality of Life (UW-QOL) Questionnaire and mouth-opening degrees before and after surgery were routinely investigated. The UW-QOL Questionnaire version 4 is a self-evaluation questionnaire for patients with the head and neck cancer, which has 12 specific question items. Each item was scored by Likert score method from 0 to 100. The higher scores represented the better quality of life. The average score of each item was obtained to compare the quality of life between the two groups. In the present study, we only included 9 best fit items (pain, appearance, neck movement, swallowing, chewing, speech, shoulder movement, taste and saliva) for our study. All data were collected by Mrs. Lu alone to avoid any bias caused by different operators.

Lower lip movement, sensation and appearance were scored from 0 to 5 using an author-developed questionnaire (Supplementary Table 1) by the patients 6 months postoperatively. All scores were calculated to assess the differences between two groups.

Patients were reviewed every 3 months to examine whether there was local and/or regional recurrence or distant metastasis by clinical examination, computed tomography, and magnetic resonance imaging. If a patient died or recurrence was observed, the survival data of the patient were censored. The overall survival (OS) and disease-free survival (DFS) times were recorded.

### Statistical analysis

Student’s *t*-test was used to compare differences in continuous variables, Mann-Whitney U test was used to assess differences in scores, chi-squared test or Fisher’s exact test was used to compare differences in categorical variables, and the Kaplan-Meier method was used to calculate survival and local control data. All statistical analyses were performed using SPSS 20.0 and Graph Pad 8.0, with a significance level of *P* < 0.05.

## Results

The demographic and clinical information of the participants is presented in Table 1. The age and sex of the patients, tumor size, T and N classification, and TNM stage were not significantly different between the two groups. Only the total operative time in the lateral approach group was significantly longer than that in the conventional approach group (*P* = 0.000). The number of harvested lymph nodes was not significantly different at each neck level, including levels I–VI. Other intraoperative and postoperative parameters were not significantly different between the two groups.

**Table 1:** Clinical and pathologic characteristics

Parameter	LA group *n* = 37	CA group *n* = 43	*P* value
Age (year)	48.8±10.0	47.8±7.9	.186
Gender			.477
Male	30 (81.1%)	32 (74.4%)	
Female	7 (18.9%)	11 (25.6%)	
T classification			.910
T1/T2	33 (89.2%)	38 (88.6%)	
T3/T4	4 (10.8%)	5 (11.6%)	
N classification			.927
N0/N1	22 (59.5%)	26 (60.5%)	
N2/N3	15 (40.5%)	17 (39.5%)	
TNM stage			.752
I/II	27 (72.9%)	30 (69.8%)	
III/IV	10 (27.1%)	13 (30.2%)	
Neck dissection			.927
MRND	15 (40.5%)	17 (39.5.0%)	
SND	22 (59.5%)	26 (60.5%)	
Bone resectoin			
Maxillectomy	8 (21.6%)	5 (11.6%)	0.352
Mandibulectomy	14 (37.8%)	21 (48.8%)	0.565
Both	5 (13.5%)	6 (13.9%)	0.723
Median follow up (month)	21	28	0.331
Operative time (min)	373±45	352±49	.000^*^
Blood loss (mL)	241±142	224±112	.563
Surgical margin (n)			
Positive/negative	0/37	0/43	NA
LN retrieved (n)			
Level I	8.81±4.34	9.40±4.80	.817
Level II	7.24±1.46	7.16±1.61	.572
Level III	7.38±4.15	8.33±3.52	.273
Level IV	6.30±2.85	5.84±2.68	.799
Level V	4.97±3.12	4.84±2.79	.838
LOHS (days)	12.1±3.6	11.7±5.5	.737
Complications (n, %)			
Salivary fistula	6 (16.2%)	8 (18.6%)	0.779
Hematoma	4 (10.8%)	5 (11.6%)	0.647
Chyle leakage	0	2 (4.7%)	0.183
Seromas	3 (8.1%)	4 (9.3%)	0.381
Flap failure	3 (8.1%)	3 (6.9%)	0.848
Wound infection	7 (18.9%)	6 (13.9%)	0.548
Facial palsy	35 (94.6%)	40 (93.1%)	0.127
Recurrence (n, %)			
Local	4 (10.8%)	6 (13.9%)	0.939
Regional (Level I)	3 (8.1%)	4 (9.3%)	0.850

*LA* Lateral approach, *CA* Conventional approach, *NA* Not applicable, *LN* Lymph nodes, *LOHS* Length of hospital stay

^*^ *P*<0.05

**Fig. 5:**
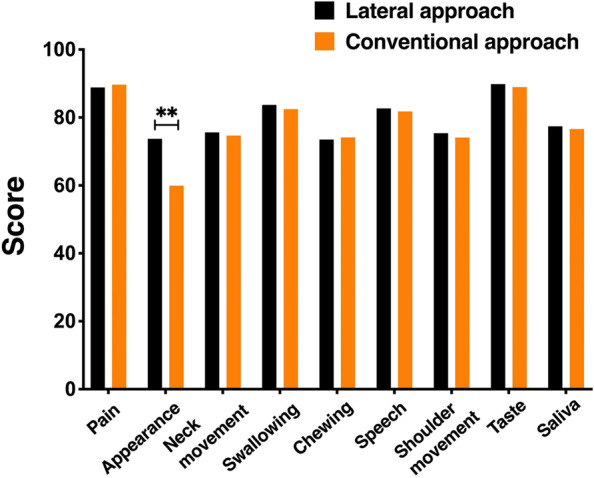
Aesthetic and functional items of UW-QOL questionnaire for two groups six months after operations

**Fig. 6:**
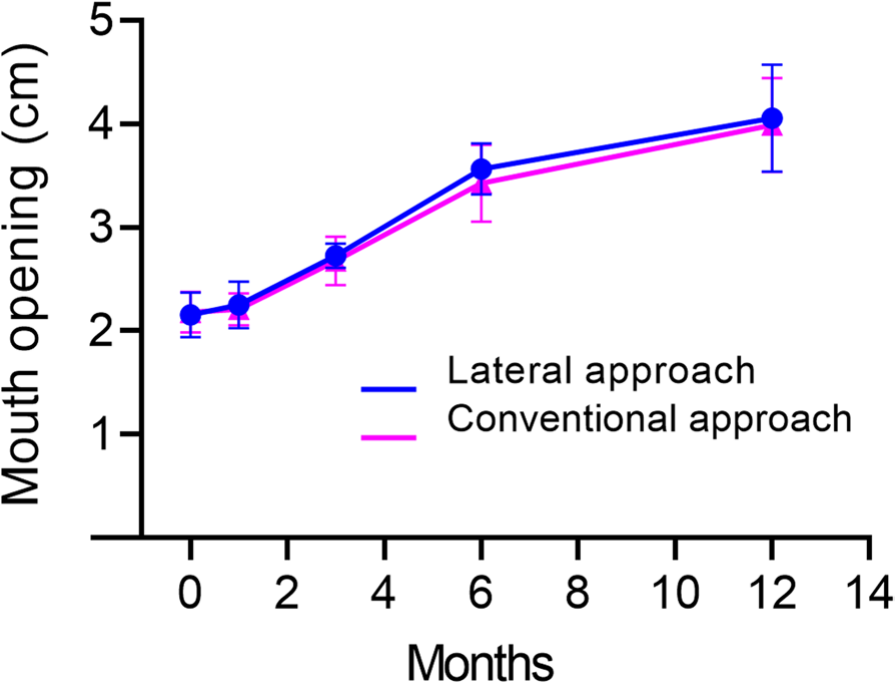
The postoperative changes of mouth opening showed an obvious tendency for mouth-opening improvement in both groups

In our UW-QOL questionnaire, the facial appearance score in the lateral approach group was significantly higher than that in the conventional approach group (*P* = 0.000), as shown in Fig. 5 (data in Supplementary Table 2). This result indicated that patients in the lateral approach group were more satisfied with their postoperative facial appearance than those in the conventional approach group. Mouth opening in the two groups showed an obvious tendency to improve from 2 to 12 months postoperatively, but no statistical difference was found between the two groups (Fig. 6, data in Supplementary Table 3).

**Fig. 7:**
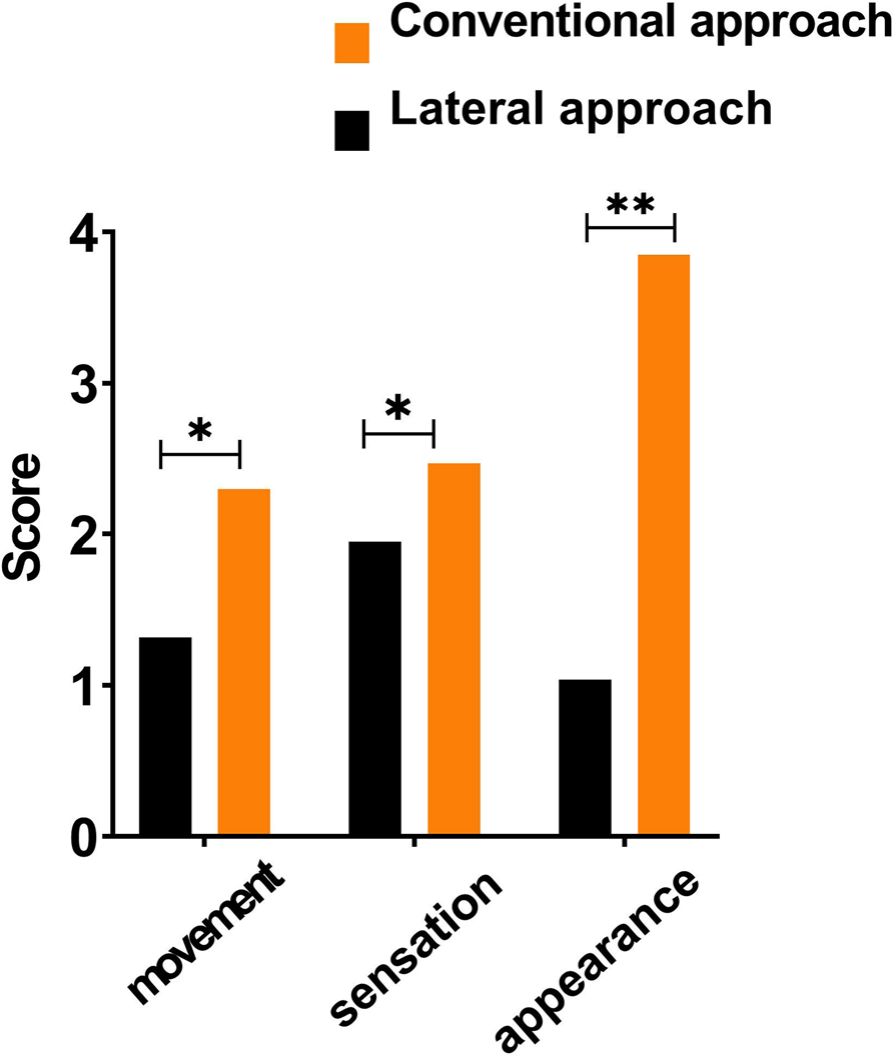
Postoperative assessment for the movement, sensation, and appearance of the lower lip in two groups

As shown in Fig. 7 (data in Supplementary Table 4), the scores of postoperative lower lip movements (*P* = 0.017), sensation (*P* = 0.035), and appearance were all significantly lower in the lateral approach group, which indicated that the conventional approach could damage the function, sensation, and appearance of the lower lip.

**Fig. 8:**
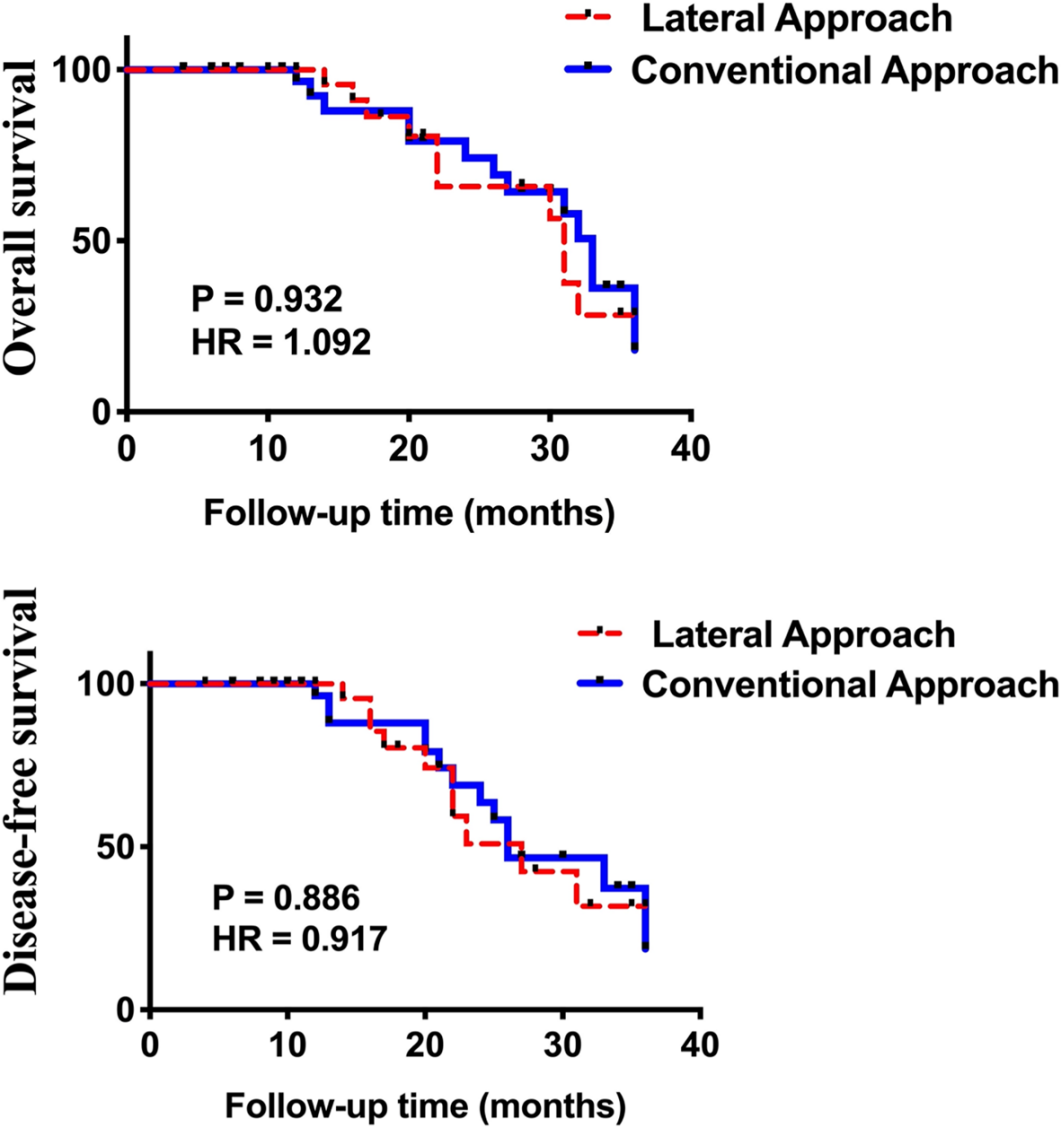
Kaplan-Meier analysis depicts no significant difference of 3-year OS and DFS between two groups

As depicted in the details of recurrences in Table 1, four patients (10.8%) developed local recurrences and three patients (8.1%) developed level I regional recurrence in the lateral approach group. In the conventional approach group, six patients (13.9%) had local recurrence and four (9.3%) had level I regional recurrence. There was no significant difference in locoregional recurrence between the two groups. Eventually, 15 patients were lost during the follow-up, and 65 patients (30 cases in the lateral approach group, and 35 in the conventional approach group) were regularly monitored for a median 28.5 ± 9.98 months and a maximum of 36 months. Kaplan-Meier analysis also showed no significant difference in the 3-year OS and DFS between the two groups (Fig. 8).

## Discussion

Lower lip-splitting incisions have been widely used to facilitate the exposure of the oral, oropharyngeal, and parapharyngeal spaces [3]. However, incisions can result in facial scars and damage to oral function. With the constant development of minimally invasive surgery, surgeons have been encouraged to improve various surgical approaches with the aim of concealing scars in the face and neck area, thereby demonstrating the interest of both surgeons and patients in cosmetic results. McGregor et al. [9] modified the lower lip-splitting approach to reduce muscle fiber disruption and scar contracture; however, it is not yet an ideal incision technique. Robson et al. [10] used the lateral lip-splitting approach for the removal of intraoral malignant tumors, which still pass across the lateral side of the lower lip and sometimes result in a hypertrophic scar under the vermilion border. Sun et al. [11] applied this lateral lip-splitting technique to maxillectomy and obtained satisfactory aesthetic results by careful closure of the vermilion, orbicularis oris muscle, and skin. However, all lip-splitting incisions are inevitably associated with scars in the face and lip deformities because of the disruption of the orbicularis oris muscle fibers [12]. Thus, some attempts have been made to avoid splitting of the lower lip when resecting intraoral tumors. Li et al. [13] used a visor approach for total or subtotal glossectomy and reconstruction, avoiding lip-splitting and mandibulotomy. He concluded that lip splitting was unnecessary for resection and reconstruction of oral cancer. In addition, Benjamin et al. [14] found that both lip-splitting and the visor flap approach could provide equally favorable exposure. It is easy to assume that less scarring results from no splitting of the lower lip. However, lower lip scars sometimes do not greatly influence patients’ awareness of the quality of life, and their major concern comes from the damage to oral function [15].

In the present study, we for the first time modified a lateral approach that was combined with facelift and hockey-stick incisions for radical resection of BSCC and compared the surgical, functional, cosmetic, and oncological outcomes between the lateral and conventional approaches. Our results showed that, on the one hand, the lateral approach can provide equally sufficient surgical exposure as the conventional approach; on the other hand, the lateral approach made postoperative scars more concealing and led to better postoperative patient satisfaction. More importantly, we found that the lateral approach resulted in superior movement, sensation, and appearance of the lower lip, although it did not affect the overall function of the oral cavity. Mouth opening, one of the most serious complications in patients, was not significantly different between the two groups. Six months after the operation, patients in the lateral approach group were more satisfied with their facial aesthetics and oral function.

Facelift incision can provide sufficient surgical visualization and good aesthetic outcomes by concealing scars of postauricular and hairline incisions [16]. Thus, parotidectomy is routinely performed via face-lift incisions to improve cosmetic effects [17]. Moreover, the face-lift approach has also been presented for removing mid-cheek masses in a plane superficial to the parotid fascia [18]. In the present study, we modified the face-life incision from the sideburn and along the anterior line of the tragus to achieve sufficient forward extension even to the corner of the mouth, which could ensure safe removal of the anterior buccal carcinoma.

Neck dissection techniques have evolved towards minimally invasive and less visible approaches while preserving oncological goals. The hockey stick incision is commonly used for neck dissection in patients with thyroid carcinoma and can provide excellent access even to neck levels I and II [19]. To perform en bloc ablation of the tumor and neck lymph tissues, we connected the postauricular end of the modified face-lift incision with the upper end of the hockey stick incision instead of the conventional submandibular incision. This design can also provide good surgical visualization of the upper and forward neck levels. To obtain better aesthetic outcomes, the horizontal line of the hockey incision could either be a transverse incision above the clavicle or along the natural skin crease of the neck, both of which lie in an inconspicuous place. Moreover, oncological control should not be overshadowed when improving the facial and neck cosmesis. We found that the number of lymph nodes retrieved was not statistically significantly different between the two approaches, which demonstrated that the oncological effectiveness of the lateral approach was not inferior to that of the conventional approach. The 3-year survival analysis also proved this conclusion. However, the ultimate effectiveness should be based on a longer follow-up period and larger number of patients.

We proposed that there are three major advantages of the lateral approach in this study: first, no incision was required to be made in the face, reducing the awareness of face and lower lip scars and increasing postoperative satisfaction; second, the appearance and function of the lower lip were preserved well because the integrity of the lower lip was not damaged, unless a part of the lower lip had to be resected due to the location of the tumor in some cases; and third, this lateral approach could dissect all concerned levels of neck dissection even if the frontmost level was Ia, indicating that such an approach did not compromise surgical safety when improving cosmetic outcomes.

However, the lateral approach still has several disadvantages. First, the total operative time in the lateral approach group was significantly longer. A longer operating time is reasonable because of the unskilled exposure and dissection, as well as the intraoral resection of the tumor and suture of the flap. Nevertheless, the difference between the two groups in operating time was only 20 min, and the clinical impact of such differences would not be significant from both the surgeons’ and patients’ perspectives. Second, this approach is not suitable for advanced buccal cancer, which requires through-and-through resection of the cheek. We believe that postoperative cosmesis of the head and neck is no longer a major concern for surgeons and patients after a full-thickness resection of the cheek. Thus, the majority of the cases were of T_1_ or T_2_ tumors, which did not require through-and-through resection of the cheek. Third, the facial nerve, either the marginal branch or the buccal branches, was likely damaged during en bloc resection using the lateral approach. However, scarification of the marginal branch is commonly inevitable in the radical treatment of buccal cancer, regardless of the approach used according to the principle of tumor resection.

This is the first study to compare the aesthetic results of the two approaches used for radical resection of BSCC. However, there are still two main limitations in this study. Firstly, cosmetic outcomes can be influenced by many factors such as operator experience, wound closure technique, and postoperative scar management. These may act as critical confounding factors when evaluating the true impact of the incision approach on postoperative cosmetic outcomes. Secondly, we only included the two approaches performed in our single institution. Different surgical teams in multiple institutions and more cases in the lateral approach group should be included to verify our results in the further study.

## Conclusion

In conclusion, this study showed that the lateral approach was feasible for the combined radical resection of BSCC and yields improved cosmetic and functional results. This led to better patient satisfaction without compromising oncological safety, although the lower-lip splitting incision is still the standard approach for the resection of BSCC.

## Supplementary Information

**Additional file 1:** **Supplementary Figure.**Twoadvanced BSCC cases undergoing the through-and-through resection of the cheekby the conventional approach (**A**) and the lateral approach (**B**).

**Additional file 2:** **Supplementary Table 1.** Questionnaire for lower lip movement, sensation, and appearanceassessment. **Supplementary Table 2.** Mean scoresof UW-QOL version 4 from patients in the two groups 6 months postoperatively.**Supplementary Table 3.** Mouth opening evaluation of patients in the twogroups before and after surgery. **Supplementary Table 4.** Postoperativeevaluation of lower-lip movement and sensation in the two groups.

## Data Availability

The datasets generated and analyzed during the current study are not publicly available due to the requests of hospitals but are available from the corresponding author on reasonable request.

## Abbreviations

BSCC: Buccal squamous cell carcinoma
LOHS: Length of hospital stay
SND: Selective neck dissection
MRND: Modified radical neck dissection
UW-QOL: University of Washington Quality of Life
OS: Overall survival
DFS: Disease-free survival
