# Supplementary material for: Lateral approach is a more aesthetical option for radical resection of BSCC: assessment of its surgical, oncological, functional, and aesthetic outcomes
Source: BMC Oral Health. 2022 Nov 3;22:464. doi: 10.1186/s12903-022-02519-1 (PMC9632109; doi:10.1186/s12903-022-02519-1)
Supplement: Supplementary file 1 — Additional file 1: Supplementary Figure.Twoadvanced BSCC cases undergoing the through-and-through resection of the cheekby the conventional approach (A) and the lateral approach (B). [file 12903_2022_2519_MOESM1_ESM.docx]

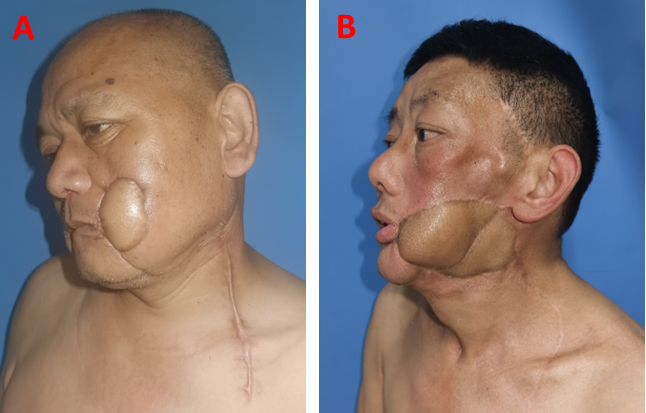


**Supplementary Figure.** Two advanced BSCC cases undergoing the through-and-through resection of the cheek by the conventional approach **(A)** and the lateral approach **(B)**.
