# Supplementary material for: Lateral approach is a more aesthetical option for radical resection of BSCC: assessment of its surgical, oncological, functional, and aesthetic outcomes
Source: BMC Oral Health. 2022 Nov 3;22:464. doi: 10.1186/s12903-022-02519-1 (PMC9632109; doi:10.1186/s12903-022-02519-1)
Supplement: Supplementary file 2 — Additional file 2: Supplementary Table 1. Questionnaire for lower lip movement, sensation, and appearanceassessment. Supplementary Table 2. Mean scoresof UW-QOL version 4 from patients in the two groups 6 months postoperatively.Supplementary Table 3. Mouth opening evaluation of patients in the twogroups before and after surgery. Supplementary Table 4. Postoperativeevaluation of lower-lip movement and sensation in the two groups. [file 12903_2022_2519_MOESM2_ESM.docx]

**Supplementary Table 1. Questionnaire for lower lip movement, sensation, and appearance assessment**

Name: Gender: Female/Male Patient No. Surgical approach: Lateral/conventional

| Parameter | Score | | | | | |
| --- | --- | --- | --- | --- | --- | --- |
|  | not affected | slightly and negligibly affected | obviously but unfrequently affected | frequently but bearably affected | sometimes unbearably affected | totally unbearably affected |
| Lower lip movement | 0 | 1 | 2 | 3 | 4 | 5 |
| Lower lip sensation | 0 | 1 | 2 | 3 | 4 | 5 |
| Lower lip appearance | 0 | 1 | 2 | 3 | 4 | 5 |

**Supplementary Table 2.** **Mean scores of UW-QOL version 4 from patients in the two groups 6 months postoperatively**

| Group | Pain | Appearance | Neck movement | Swallowing | Chewing | Speech | Shoulder movement | Taste | Saliva |
| --- | --- | --- | --- | --- | --- | --- | --- | --- | --- |
| Lateral Approach  (n＝36) | 88.89 | 73.75 | 75.61 | 83.72 | 73.53 | 82.69 | 75.39 | 89.86 | 77.42 |
| Conventional approach  (n＝40) | 89.68 | 59.93 | 74.68 | 82.48 | 74.13 | 81.78 | 74.1 | 88.95 | 76.58 |
| *P* value | 0.474 | **0.000^*^** | 0.624 | 0.632 | 0.913 | 0.566 | 0.431 | 0.465 | 0.552 |

^*^ *P*＜0.05

**Supplementary Table 3. Mouth opening evaluation of patients in the two groups before and after surgery**

| Time | Lateral approach group  (cm, mean±SD) | Conventional approach group  (cm, mean±SD; n) | *P* value |
| --- | --- | --- | --- |
| Preoperative | 2.14±0.74 | 2.18±0.67 | 0.528 |
| 1 month postoperatively | 2.28±0.55 | 2.24±0.54 | 0.744 |
| 3 months postoperatively | 2.73±0.44 | 2.71±0.52 | 0.235 |
| 6 months postoperatively | 3.56±0.30 | 3.42±0.38 | 0.315 |
| 12 months postoperatively | 4.05±0.21 | 3.99±0.44 | 0.813 |

**Supplementary Table 4. Postoperative evaluation of lower-lip movement and sensation in the two groups**

| Parameter | Lateral approach group  n =36 | Conventional approach group  n = 40 | *P* value |
| --- | --- | --- | --- |
| Lower lip movement | 1.32±0.94 | 2.30±1.02 | 0.017^*^ |
| Lower lip sensation | 1.95±1.06 | 2.47±0.96 | 0.035^*^ |
| Lower lip appearance | 1.04±0.29 | 3.85±0.22 | 0.00^*^ |

^*^ *P*＜0.05
